# Integrated omics analysis of coronary artery calcifications and myocardial infarction: the Framingham Heart Study

**DOI:** 10.1038/s41598-023-48848-1

**Published:** 2023-12-07

**Authors:** Amalie Lykkemark Møller, Ramachandran S. Vasan, Daniel Levy, Charlotte Andersson, Honghuang Lin

**Affiliations:** 1 Section of Preventive Medicine and Epidemiology, Department of Medicine, Boston University School of Medicine, Boston, MA USA; 2 Department of Cardiology, Nordsjællands Hospital, Hillerød, Denmark; 3 Boston University’s and National Heart, Lung, and Blood Institute’s Framingham Heart Study, Framingham, MA USA; 4 University of Texas School of Public Health San Antonio, and Departments of Medicine and Population Health Sciences, University of Texas Health Science Center, San Antonio, TX USA; 5 Population Sciences Branch, Division of Intramural Research, National Institutes of Health, Bethesda, MD USA; 6 Section of Cardiovascular Medicine, Department of Medicine, Boston Medical Center, Boston University School of Medicine, Boston, MA USA; 7 Department of Medicine, University of Massachusetts Chan Medical School, Worcester, MA USA

**Keywords:** Clinical genetics, Genomics, Cardiology

## Abstract

Gene function can be described using various measures. We integrated association studies of three types of omics data to provide insights into the pathophysiology of subclinical coronary disease and myocardial infarction (MI). Using multivariable regression models, we associated: (1) single nucleotide polymorphism, (2) DNA methylation, and (3) gene expression with coronary artery calcification (CAC) scores and MI. Among 3106 participants of the Framingham Heart Study, 65 (2.1%) had prevalent MI and 60 (1.9%) had incident MI, median CAC value was 67.8 [IQR 10.8, 274.9], and 1403 (45.2%) had CAC scores > 0 (prevalent CAC). Prevalent CAC was associated with AHRR (linked to smoking) and EXOC3 (affecting platelet function and promoting hemostasis). CAC score was associated with VWA1 (extracellular matrix protein associated with cartilage structure in endomysium). For prevalent MI we identified FYTTD1 (down-regulated in familial hypercholesterolemia) and PINK1 (linked to cardiac tissue homeostasis and ischemia–reperfusion injury). Incident MI was associated with IRX3 (enhancing browning of white adipose tissue) and STXBP3 (controlling trafficking of glucose transporter type 4 to plasma). Using an integrative trans-omics approach, we identified both putatively novel and known candidate genes associated with CAC and MI. Replication of findings is warranted.

People with a first-degree relative with myocardial infarction (MI) have a two- to four-fold increased relative risk of developing the disease^1^, indicating a significant genetic role in disease development. Multiple genetic variants have been identified for coronary artery disease^2,3^. Still, identified variants have been found to explain less than 15% of the heritability, and familial coronary artery disease remains an independent predictor of coronary disease after adjusting for known common genetic variants^2–4^, underscoring that additional approaches are needed to identify the residual genetic variation. Identification of biological pathways involved in coronary artery calcification (CAC) and MI has the potential to pinpoint novel therapeutic approaches to prevent disease occurrence and progression.

Previous population-based genomic studies have largely analyzed each trans-omic data type separately and have applied very stringent statistical cutoffs to reduce false-positive associations. However, this comes at a cost of low sensitivity for capturing true positive findings. With an integrative analysis of a range of different omics components, directionally concordant associations will reduce the risk of both false-positive and false-negative findings^5^. By applying this method for heart failure and echocardiographic traits, we have previously identified several plausible genetic variants associated with these outcomes^6^. Although conventional genomic association methods have yielded more genetic variants for coronary disease compared with heart failure, we postulated that more candidate genes could be identified for coronary disease using similar methods. The aim of this study was, therefore, to search for additional genetic loci associated with CAC and MI by integrating associations found across GWAS, DNA methylation, and gene expression.

## Methods

### Population

This study included participants from the Framingham Heart Study’s (FHS) Offspring and Third Generation cohorts. Detailed descriptions of the cohorts are available elsewhere^7,8^. Individuals were included if they had data on at least one of the omics of interest. In the case an individual did not have data on all omics, they only contributed to the omics analysis for which they had data. We followed patients from the date of the genomic profiling (date of the blood sample) until December 31st, 2016, or until MI or death, whichever occurred first.

### Omics measures

Blood samples were collected during 1998–2008 for the Offspring cohort (examination cycle 7 and 8), and 2002–2005 for the Offspring Spouse and Third generation cohort (1st examination cycle).

Affymetrix 550 k Array (Affymetrix, Santa Clara, CA) was used for profiling of genetic variants, which were then imputed to the 1000 Genomes Project by MaCH (v 1.0.15)^9^ and only variants with an imputation quality greater than 0.3 were retained. DNA methylation was available for participants in the Offspring cohort from 8th examination cycle and Third generation cohort from 2nd examination cycle. The profiles for DNA methylation were measured from whole blood derived DNA using the Infinium HumanMethylation450 BeadChip (Illumina, San Diego, CA)^10^. Rigorous quality control was performed and only high quality CpG sites were kept. The gene expression profiling was derived from isolated RNAs from fasting peripheral whole blood on the Affymetrix Human Exon 1.0st Array (Affymetrix, Santa Clara, CA) for the Offspring cohort and Third Generation cohort at the same exams at which DNA methylation was assessed. More details of the methods for the profiling of omics data is available in previous publications^6,11^.

### Outcomes

To investigate associations between gene function and coronary disease development and progression, respectively, a total of four outcomes were analyzed: CAC as a dichotomous variable (presence or absence of CAC), CAC as a continuous variable (excluding those with a CAC score of 0), prevalent MI, and incident MI. Separate analyses were undertaken for prevalent and incident MI to avoid incorrect handling of time (since pooling prevalent and incident cases may lead to spurious associations in opposite directions). The two measures of CAC were chosen to allow for investigations of associations between gene function and (1) development of calcification (CAC as a dichotomous variable) and (2) the extent of the calcification among subjects with CAC (CAC as a continuous variable). MI was diagnosed by ECG, enzymes and history, or autopsy evidence. CAC prevalence was estimated from coronary tomography (CT) scans undertaken for the Offspring in 1998–2001 (7th examination cycle) and Third generations in 2002–2005 (1st examination cycle), and the extent of CAC was quantified by the Agatston score^12^. The Agatston score is computed by multiplying the lesion area with a weighted attenuation score (based on the maximal attenuation score within the lesion), where a calcified lesion is an area including at least 3 connected pixels with CT attenuation > 130 Hounsfield units^13^.

### Statistics

The analysis was performed in three steps. In the first step each omics measure (GWAS, DNA methylation, and gene expression) was regressed to each of the four outcomes. Given the familial relatedness among FHS participants, generalized estimating equations were used to test the association of omics measures with the presence of CAC and prevalent MI. Similarly, linear mixed models were used to assess the association between omics measures and CAC values. In addition, Cox proportional hazards models clustering on pedigrees were used to assess the association between omics measures and incident MI. All models were adjusted for age, sex, weight, height, and technical covariates.

In the second step, the associations of each of the four outcomes with each type of omic measures (GWAS, DNA methylation, and gene expression) were summarized at the gene level. For the genetic associations, the most significant genetic variant within each gene region was used to represent the overall association of the gene with the outcomes. Similarly, the most significant CpG site within each gene region was used to represent the overall association of methylation profile with the outcomes. For the gene expression, the most significant transcript was used to represent the association of each gene with the outcomes. Finally, we used robust rank aggregation to integrate the top 5% association from the three different omic data types. It tested how much better the gene was positioned in the ranked list than what would be expected by chance, which is formalized by randomly shuffling of the ranked list^5^. A trans-omic score was calculated to represent the significance of each gene across the different omic data types. A full description of the statistical test is available elsewhere^5^. The test results from the top 10 genes with the lowest trans-omic scores are shown for each outcome. Additionally, we highlighted genes that were identified among the top genes with the trans-omic scores for more than one of the studied outcomes. All trans-omic scores and the *p*-values from the individual analyses are available in Tables S1, S2, S3, and S4 in the Supplemental Data, which may be used by other researchers as a reference.

### Ethics approval and consent to participate

The present study was approved by the Institutional Review Board of the Boston Medical Campus. A written informed consent has been collected from all individuals prior to entering the FHS. The study was conducted according to the Declaration of Helsinki.

## Results

As shown in Table 1, the current study included 3106 participants with CAC measurements. The mean age was 57 years and 48.9% were female. In total, 1403 (45.2%) had CAC, and the median CAC value was 67.8 (IQR 10.8, 274.9). We found 65 individuals with prevalent MI and 60 with incident MI during a mean follow-up of 8.2 years. For the GWAS analysis, we included 2932 individuals. For the analyses of the DNA methylation and gene expression we included 1936 and 2729 individuals, respectively. In Table 2, characteristics of the individuals according to the outcomes are shown. Individuals with MI or CAC were on average older, more often male, and had higher prevalence of hypercholesterolemia, hypertension, obesity, and diabetes compared to the total study population (Table 2) .

**Table 1:** Characteristics of study participants.

Characteristic/analysis	Total (n = 3106)
All analyses*	3106
GWAS, n (%)	2932 (94.4%)
Gene expression, n (%)	2729 (87.9%)
DNA methylation, n (%)	1936 (62.3%)
Age, mean (SD)	57 ± 10
Sex (women), n (%)	1519 (48.9%)
Obese (BMI above 30), n (%)	993 (32.0%)
Diabetes, n (%)	251 (8.1%)
Smoking, n (%)	244 (7.9%)
Hypercholesterolemia, n (%)	975 (31.4%)
Hypertension, n (%)	1289 (41.5%)
Prevalent CAC, n (%)	1403 (45.2%)
Continuous CAC, median IQR	67.8 (10.8, 274.9)
Prevalent MI, n (%)	65 (2.1%)
Incident MI, n (%)	60 (1.9%)

All comorbidities are measured at baseline, i.e. the time of the Framingham Heart Study examination.

*BMI* body mass index, *MI* myocardial infarction, *CAC* coronary artery calcification.

*In our study population, 2589 (83.4%) had data on both GWAS and gene expression, 1842 (59.3%) had data on both GWAS and DNA methylation, 1690 (54.4%) had data on both gene expression and DNA methylation, and 1614 (52.0%) had all data on all three omics.

**Table 2:** Characteristics of study participants according to the outcomes; presence of coronary artery calcification and myocardial infarction (prevalent and incident).

Characteristic	Outcomes		
	Presence of CAC (n = 1403)	Prevalent MI (n = 65)	Incident MI (n = 60)
Age mean (SD)	63 ± 11	67 ± 10	64 ± 10
Sex (women), n (%)	518 (36.9%)	17 (26.2%)	26 (43.3%)
Obese (BMI above 30), n (%)	532 (37.9%)	24 (36.9%)	29 (48.3%)
Diabetes, n (%)	183 (13.0%)	14 (21.5%)	10 (16.7%)
Smoking, n (%)	115 (8.2%)	10 (15.4%)	4 (6.7%)
Hypercholesterolemia, n (%)	640 (45.6%)	54 (83.1%)	30 (50.0%)
Hypertension, n (%)	834 (59.4%)	56 (86.2%)	40 (66.7%)

*CAC* coronary artery calcification, *MI* myocardial infarction, *BMI* body mass index.

### Association with coronary artery calcifications (CAC)

The top 10 most significant genes for the analyses of CAC included *TMEM80, HAPLN2, GAK, PDCD6-AHRR, AHRR, EXOC3, SLC9A3-AS1, ALAS1, DNAH1,* and *TNFRSF1A* for prevalent CAC, and *TECPR2, GABARAP, ALPI, MACROD2, TTC34, VWA1, ZNF839, MOK, CLEC4F,* and *LOC101927666* for CAC as a continuous variable. A full list of annotations, putative functions, and locations of the top 10 genes are presented in Table 3. All trans-omic scores for the three omics data for CAC presence (dichotomous) and CAC score (continuous) are available in Tables S1 and S2 in the Supplemental Data.

**Table 3:** Trans-omic scores and *p*-values for GWAS, DNA-methylation, gene expression for CAC (prevalent and continuous).

Chromo some/locus	Gene name	Trans-omic score	Genomics	Epigenomics	Transcriptomics	Annotation/function
**Prevalent CAC**						
Chr. 11 (p15.5)	*TMEM80*	2.37E−05	2.78E−05	1.00E−04	8.06E−03	Transmembrane protein 80
Chr. 1 (q23.1)	*HAPLN2*	3.66E−05	1.30E−06	5.19E−03	5.57E−03	Protein coding gene, also known as brain derived link protein 1 (Bral1). Known to bind hyaluronan, which is expressed in the progression of atherosclerotic plaques ^14,15^
Chr. 4 (p16.3)	*GAK*	9.88E−05	6.63E−05	8.84E−05	1.11E−03	Cyclin G Associated Kinase, transcriptional target of p53 tumor suppressor gene
Chr. 5 (p15.33)	*PDCD6-AHRR*	1.82E−04	3.80E−06	1.23E−05		RNA coding gene. Overlapping with AHRR and close to EXOC3
Chr. 5 (p15.33)	*AHRR*	1.84E−04	3.80E−06	1.23E−05	6.38E−01	Aryl-Hydrocarbon Receptor Repressor. Can bind to nuclear factor-kappa B (NFKB) and may be immune modulating^16^. It has previously been associated with smoking^17^. AHRR DNA methylation has also been associated with carotid intima-media thickness^18^. AHRR expression is increased in atherosclerotic lesions of mice and may in conjunction with other genes (such as TCF21) activate an inflammatory response in the coronary artery smooth vessels^19^
Chr. 5 (p15.33)	*EXOC3*	1.87E−04	3.80E−06	1.23E−05	1.12E−01	EXOC3 affects platelet function and promotes hemostasis and accelerates arterial thrombosis in mice^20^ EXOC3 expression increases glucose uptake in adipocytes. EXOC3 and AHRR is located in close proximity
Chr. 5 (p15.33)	*SLC9A3-AS1*	1.90E−04	3.80E−06	1.23E−05		Sodium proton exchanger type 3, may lead to arterial hypertension and has been suggested to be a novel target for antihypertensive medications^21^. Located close to AHRR
Chr. 3 (p21.2)	*ALAS1*	2.33E−04	3.97E−03	8.84E−06	2.28E−04	ALAS-1 is involved in the synthesis of heme in various tissues^22^. Heme can increase oxidative stress, act proinflammatory, and has previously been linked with cardiovascular disease^23^
Chr. 3 (p21.1)	*DNAH1*	2.37E−04	3.68E−03	8.84E−06	3.88E−03	Structural cilia genes^24^
Chr. 12 (p13.31)	*TNFRSF1A*	2.85E−04	1.42E−05	3.51E−04	1.01E−04	Tumor necrosis factor (TNF) Receptor type 1. A major receptor for TNF-alpha, which is closely related to atherosclerotic formation^25^. High expression levels have previously been found in atherosclerotic plaques rich in foam cells^26^
**CAC continuous**						
Chr. 14 (q32.31)	*TECPR2*	3.19E−05	4.36E−05	3.59E−05	1.70E−02	Tectonin beta-propeller repeat containing 2. Involved in autophagy^27^
Chr. 17 (p13.1)	*GABARAP*	1.30E−04	1.82E−04	5.67E−04	6.52E−03	GABA A receptor associated protein; linked to autophagic activity and possibly atherosclerotic formation^28,29^
Chr. 2 (q37.1)	*ALPI*	2.56E−04	1.31E−02	6.50E−05	2.58E−02	Intestinal alkaline phosphatase. Involved in fat absorption – knockout mice display increased fat absorption.^30^ ALP1 deficiency has previously been associated with the metabolic syndrome and an increased risk of ischemic heart disease in humans^31^
Chr. 20 (p12.1)	*MACROD2*	3.19E−04	9.60E−07	1.94E−02	6.08E−01	Mono-ADP Ribosylhydrolase 2. Previously associated with obesity and brain infarcts^32,33^
Chr. 1 (p36.32)	*TTC34*	3.19E−04	3.22E−03	1.88E−06		Tetratricopeptide Repeat Domain 34
Chr. 1 (p36.33)	*VWA1*	3.19E−04	6.81E−02	1.09E−03	7.04E−03	Von Willenbrand factor A Domain containing 1. Expression of VWA1 in mouse cardiac endothelial cells have shown to be significantly affected by obesity, ageing, and physical activity^34^
Chr. 14 (q32.31)	*ZNF839*	3.36E−04	3.16E−05	3.59E−05	7.89E−02	Zinc finger protein 839, previously implicated in chronic obstructive pulmonary disease and resting heart rate^35^ Closely located to MOK and TECPR2
Chr. 14 (q32.31)	*MOK*	3.44E−04	3.16E−05	3.59E−05		MOK protein kinase
Chr. 2(p13.3)	*CLEC4F*	5.05E−04	2.45E−02	1.09E−04	2.26E−02	C-type Lectin Domain Family 4 Member F, exclusively expressed on hepatic Kupffer cells. Hepatic expression of cholesteryl ester transfer protein (CETP) has shown to be confined to these cells^36^
Chr. 17 (q22)	*LOC10 1927666*	5.34E−04	4.68E−05	5.01E−05		Long non-coding RNA

*CAC* coronary artery calcification.

### Association with myocardial infarction (MI)

The 10 most significant genes comprised *LAT, C1orf131, FYTTD1, PPFIBP1, AKAP8, MDC1-AS1, PINK1, BRD4, TUBB, and C1orf128* for prevalent MI, and *BTF3L4, STXBP3, LINC02169, IRX3, WDR35, USP34, FOXF2, MIR6720, NDUFA11, and LOC100128568* for incident MI. Annotations, putative functions, and locations of the top 10 genes are presented in Table 4. The trans-omic scores for the three omics data for prevalent and incident MI can be found in Table S3 and S4 in the Supplemental Data.

**Table 4:** Trans-omic scores and p-values for genomics, epigenomics and transcriptomics association studies of prevalent and incident MI.

Chromosome/locus	Gene name	Trans-omic score	Genomics	Epigenomics	Transcriptomics	Annotation/Function
**Prevalent MI**						
Chr. 16 (p11.2)	*LAT*	3.83E−05	1.16E−10	3.65E−05	1.58E−02	Linker For Activation Of T Cells, linked to development and function of T cells^37^
Chr. 1 (q42.2)	*C1orf131*	8.92E−05	3.96E−09	7.36E−03	6.59E−04	Chromosome 1 Open Reading Frame 131
Chr. 3 (q29)	*FYTTD1*	1.09E−04	2.75E−05	4.20E−05	1.36E−02	Forty-two–three domain-containing protein 1. Protein Coding gene, enables RNA binding. Has been found to be down regulated among patients with familial hypercholesterolemia compared to controls^38^
Chr. 12 (p11.23-p11.22)	*PPFIBP1*	2.88E−04	7.21E−07	2.69E−06	8.59E−02	Liprin beta 1 protein coding gene
Chr. 19 (p13.12)	*AKAP8*	3.19E−04	6.04E−11	4.23E−03	4.05E−02	A-kinase anchor protein 8, enzyme that bind to protein kinase A (PKA), possibly involved in TNF-alpha signaling dependent pathways^39^ AKAP8 inhibition decreases cell apoptosis in rats ^40,41^
Chr. 6 (p21.33)	*MDC1-AS1*	3.19E−04	5.27E−05	3.62E−14		MDC1 antisense RNA 1. Long noncoding RNA
Chr. 1 (p36.12)	*PINK1*	3.19E−04	3.84E−03	3.54E−04	1.08E−02	PTEN-induced kinase 1. Mutations cause mitochondrial dysfunction and increased sensitivity to reactive oxygen species and an intact PINK1 is important for normal cardiac tissue homeostasis^42^ The loss of PINK1 have been found to increase the heart's vulnerability to ischemia–reperfusion injury^43^
Chr. 19 (p13.12)	*BRD4*	6.37E−04	6.04E−11	6.99E−04	4.66E−01	Bromodomain-containing protein 4, might be involved in several cardiovascular processes and improve endothelial integrity. It has been suggested as novel therapeutic target for prevention of cardiovascular diseases^44^ Close to AKAP8
Chr. 6 (p21.33)	*TUBB*	6.37E−04	5.27E−05	3.62E−14	8.24E−01	Tubulin beta class 1. Close to BRD4
Chr. 1 (p36.11)	*C1orf128*	6.37E−04			4.89E−03	Also known as PITHD1; has been shown to be an important activator for megakaryocyte differentiation^45^
**Incident MI**						
Chr. 1 (p32.3)	*BTF3L4*	5.52E−05	1.24E−03	5.99E−06	3.13E−02	Basic transcription factor 3-like 4 (BTF3L4), previously associated with obesity^46^
Chr. 1 (p13.3)	*STXBP3*	6.68E−05	5.20E−03	6.12E−06	1.04E−02	Syntaxin Binding Protein 3, STXBP3 is involved in the regulation of glucose uptake by controlling trafficking of Glucose transporter type 4 (GLUT4) to plasma^47^
Chr. 16 (q12.2)	*LINC02169*	1.17E−04	2.43E−08	1.59E−06		Long Intergenic Non-Protein Coding RNA 2169
Chr. 16 (q12.2)	*IRX3*	1.19E−04	2.43E−08	1.59E−06	8.28E−01	Iroquois homeobox 3 enhance browning of white adipose tissue and has been shown to be associated with obesity^48,49^
Chr. 2 (p24.1)	*WDR35*	1.46E−04	3.67E−04	3.75E−06	1.03E−02	WD repeat-containing protein 35. The TTC32-WDR35 gene cluster have been found associated with CAD. SNPs at this gene cluster might contribute to variation of HDL levels and could affect CAD severity^50^
Chr. 2 (p15)	*USP34*	2.22E−04	2.13E−05	3.34E−04	1.86E−02	Ubiquitin Specific Peptidase 34
Chr. 6 (p25.3)	*FOXF2*	2.43E−04	5.85E−09	1.88E−05	3.84E−01	FOXF2 encodes Forkhead Box F2. FOXF2 has previously been associated with stroke risk in a GWAS study^51^
Chr. 6 (p25.3)	*MIR6720*	2.46E−04	5.85E−09	1.88E−05		MicroRNA 6720, positioned close to FOXF2
Chr. 19 (p13.3)	*NDUFA11*	2.63E−04	2.82E−09	1.94E−05	7.35E−01	NADH Dehydrogenase (Ubiquinone) 1 Alpha Subcomplex 11, has been associated with heart rate variability^52^
Chr. 19 (p13.3)	*LOC100128568*	2.67E−04	2.82E−09	1.94E−05		Long non-coding RNA

*MI* myocardial infarction.

### Integration of different outcomes

**Figure 1:**
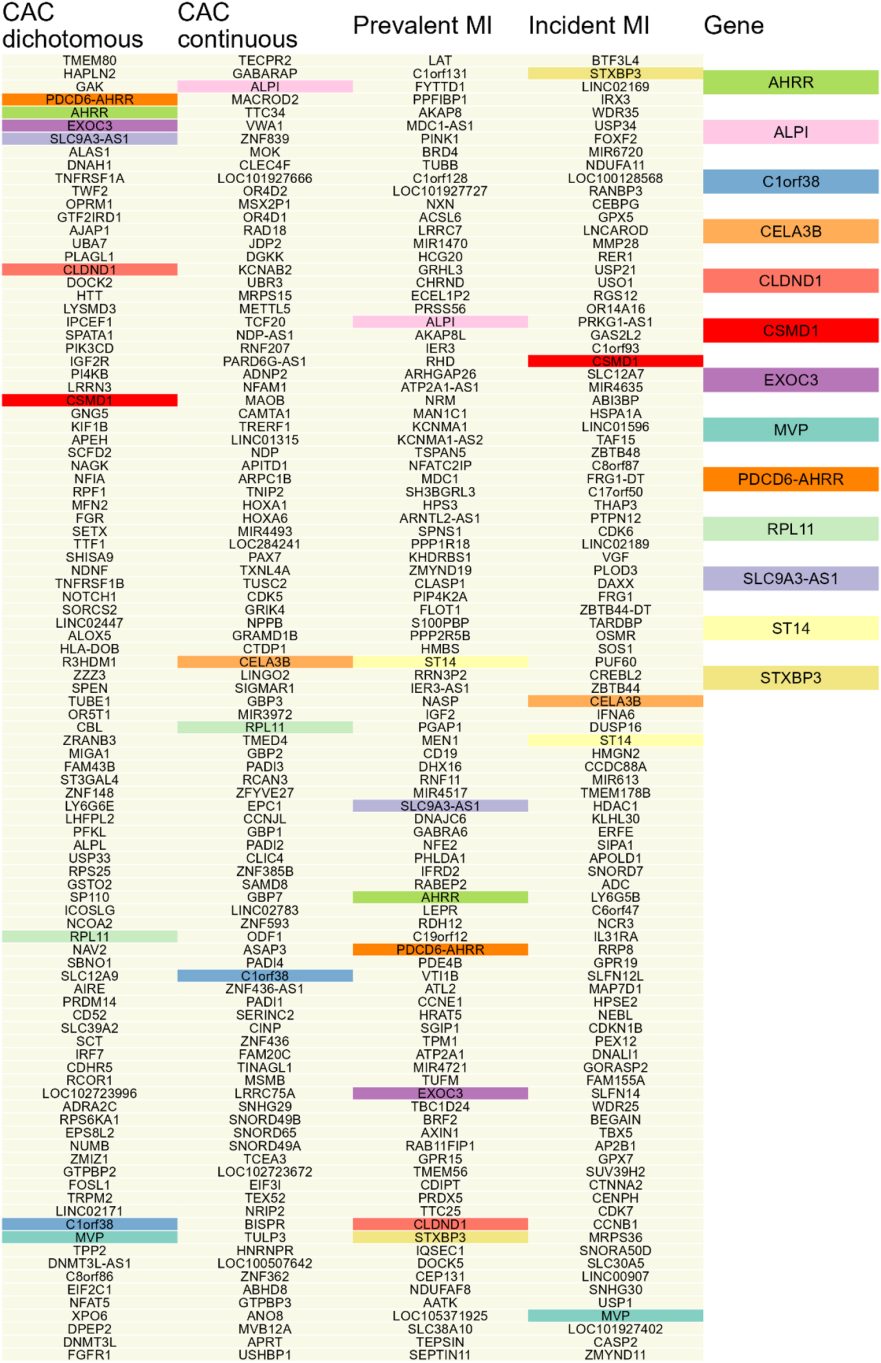
Top 100 genes for each outcome with the lowest trans-omic scores. Genes identified in top 100 for more than one outcome was highlighted.

We finally compared the list of the top 100 genes with the lowest trans-omic scores for each outcome to each other and identified genes matches (Fig. 1). In total, 13 genes were found to associate with more than one of the outcomes. These genes included PDCD6-AHRR, AHRR, EXOC3 and SLC9A3-AS1 (Table 5). We further examined the enrichment of top genes in biological pathways, and found that the top enriched pathways include basal transcription factors, estrogen signaling pathway, and longevity regulating pathway, suggesting potential functions of these biological pathways in pathology of MI.

**Table 5:** Name, location, and annotation/function of genes identified in top 100 for at least two outcomes.

Chromosome/locus	Gene name	Annotation/Function
Chr. 1 (p35.3)	*C1orf38*	Chromosome 1 open reading frame 38, also known as THEMIS2. Implicated in macrophage inflammatory response^53^
Chr. 1 (p36.12)	*CELA3B*	Chymotrypsin Like Elastase 3B. Cholesterol binding protein with proteolytic properties^54^
Chr. 3 (q11.2)	*CLDND1*	Claudin Domain Containing 1. Methylation has been associated with triglyceride levels and body mass index^55^
Chr. 8 (p23.2)	*CSMD1*	CUB And Sushi Multiple Domains 1
Chr. 16 (p11.2)	*MVP*	Major Vault Protein (MVP) is important to NF-KB signaling constraint. MVP gene knockout in mice exacerbate obesity, insulin resistance, hepatic steatosis and atherosclerosis^56^
Chr. 1 (p36.11)	*RPL11*	Ribosomal Protein L11. Inhibits peroxisome proliferator-activated receptor alpha (PPARa) ^57^, which is strongly implicated in atherosclerosis^58^
Chr. 11 (q24.3)	*ST14*	Matriptase, also known as PRSS14/Epithin. Involved in transendothelial migration of activated macrophages^59^
Chr. 5 (p15.33), Chr. 2 (q37.1), and Chr. 1 (p13.3)	*PDCD6-AHRR, AHRR, EXOC3, SLC9A3-AS1, ALPI, and STXBP3*	See Tables 3 and 4 for gene annotation/function

The table includes 13 genes that were identified in the top 100 of lowest rank-scores for at least two of the outcomes considered. All the top 100 genes for each outcome can be found in Fig. 1, where the 13 genes identified for more than one outcome are highlighted.

## Discussion

In this analysis, we integrated the association results from three omics data (GWAS, DNA methylation, and gene expression) to identify molecular signatures related to CAC and MI. We also provided a full list of genes from the analyses in the online material allowing other researchers to access all our results. It is important to point out that the present study did not have any formal cutoff to claim statistical significance and the results from this and prior studies are therefore not directly comparable. In this context, those top loci did not reach the conventional genome-wide significance cutoff. For many of the top ranked genetic loci, there are other levels of evidence suggesting that they may be involved in the pathogenesis of coronary disease, as discussed in the next sections, which also aligns with pathophysiological pathways of atherosclerosis identified in previous studies^60^.

Among the top 10 genes associated with CAC levels (excluding those with no CAC), there were 4 genes located in proximity to each other at chromosome 5 (PDCD6-AHRR, AHRR, EXOC3, and SLC9A3-AS1). Aryl-Hydrocarbon Receptor Repressor (AHRR), which can bind to nuclear factor-kappa B (NFKB) and may be immune modulating^16^, has previously been reported to be upregulated among smokers. Further, variation in DNA methylation in the AHRR gene has previously been associated with carotid plaque scores, even after adjustment for smoking status^17^. The AhR pathway, which can be activated by smoking, can increase the expression of inflammatory markers in macrophages and is involved in the buildup of lipids in macrophages and formation of plaque^17,61^. EXOC3 is important for controlling granule secretion and glycoprotein receptor trafficking in platelets, and in EXOC3 conditional knockout mice arterial thrombosis was found to be accelerated along with improved homeostasis^20^. The sodium proton exchanger subtype 3 (SLC9A3) is highly expressed in the small intestine and colon, where it absorbs salt in the gastrointestinal tract and affects the extracellular fluid volume and blood pressure. SLC9A3 is a potential drug target for hypertension by reducing salt uptake in the gut^21^. These genes were also among the top genes across all outcomes.

As expected from what we know of vascular biology, several of the top genes are known to be involved in inflammation, macrophage signaling, and endothelial function. Neither of these genes have, however, been firmly identified by GWAS previously. For instance, HAPLN2 binds hyaluronan, which is expressed in relation to inflammatory signaling and appears to be involved in the progression of atherosclerotic plaques, was among the top genes for the CAC presence^14,62^. The tumor necrosis factor (TNF) receptor type 1 (prevalent CAC), A-kinase anchor protein 8 (an enzyme that bind to protein kinase A, prevalent MI), and Cyclin G Associated Kinase (a transcriptional target of p53 tumor suppressor gene, prevalent CAC), appear all to be downstream targets of the TNF-alpha signaling pathways^39^. ST14 (Matriptase, also known as PRSS14/Epithin) represent another potentially interesting pathway that may relate to macrophage migration into the arterial walls. It has previously been reported to be involved in the transendothelial migration of activated macrophages^59^.

Moreover, several genes have previously been implicated in lipid metabolism, including ALP1, which is involved in intestinal fat absorption^30^. ALP1 deficiency is linked to the metabolic syndrome and ischemic heart disease in humans^31^. CLEC4F, identified for continuous CAC, may be directly involved in cholesteryl ester transfer protein (CETP) production^36^ and has been proposed as a target for CVD^63^. The BRD4 is part of the bromodomain and extra-terminal (BET) protein family^44^ and has been suggested to be of importance for integration of the endothelium. Inhibition of the BET reader protein has been suggested as a possible strategy in the prevention of adverse vascular remodeling^64^.

### Strengths and limitations

Strengths of the present study included multiple omics measures in a well-phenotyped cohort, and the familial relatedness in FHS, which could increase the likelihood of finding genetic mechanisms underlying MI given coronary disease clusters in families. We further integrated evidence from multi omics data to reduce false positive findings. Our study revealed multiple pathways possibly involved in the development of coronary disease. Our analyses should, however, be considered as hypothesis generating only. Although several pathways have been implicated in the pathogenesis of atherosclerosis and MI risk before, replication in independent cohorts would have further strengthened the plausibility of our findings. The use of whole blood to measure gene expression is a feasible, yet an imprecise measure of the actual gene activity within coronary arteries and comprise a limitation. Finally, this study includes only a moderate sample size with a very limited number of events and consists of a predominantly White population of European descent. Despite its limited sample size, the deep phenotyping, multi-omics measures, and multigenerational structure are unique features of the cohort, justifying the present set of analyses.

## Conclusion

Using a trans-omic approach we integrated data from GWAS, DNA methylation, and gene expression to identify potential biological mechanisms in the development of CAC and MI. We identified several candidate genes for MI and CAC, of which many have been implicated in prior studies. Further research is still needed to confirm our findings and identify potential pathways for the prevention and treatment of coronary artery disease.

### Supplementary Information

Supplementary Tables.

## Data Availability

Access to anonymized data is possible through the National Institutes of Health database of phenotypes and genotypes (https://www.ncbi.nlm.nih.gov/gap/).
